# TiME for a change: The tumor microenvironment as the missing piece in cancer therapeutics

**DOI:** 10.1371/journal.pbio.3003276

**Published:** 2025-07-24

**Authors:** Jeremy Mo, Hanyun Zhang, Alexander Swarbrick

**Affiliations:** 1 Cancer Ecosystems Program, Garvan Institute of Medical Research, Darlinghurst, Australia; 2 School of Clinical Medicine, Faculty of Medicine & Health, UNSW Sydney, Sydney, Australia; Princeton University, UNITED STATES OF AMERICA

## Abstract

Current anti-cancer treatments often neglect the role of the tumor microenvironment (TME) in resistance and progression. In this Perspective, the authors argue that better preclinical models, understanding of stromal heterogeneity and integration of TME assessments into drug development are needed to improve treatment efficacy.

## Teaser

Cancer treatments often fall short of durable cures, yet most therapeutic strategies neglect the tumor stroma. A concerted effort is needed to understand, model, target and reprogram the tumor ‘soil’, recognizing its profound influence on cancer from tumorigenesis to therapy resistance.

The advent of immunotherapies and molecularly targeted agents has transformed cancer treatment. Yet, for too many patients, durable responses remain elusive. The elucidation of the genetic model of cancer evolution enabled groundbreaking advances in our understanding of cancer initiation, progression and treatment. However, it also fostered a reductionist view of cancers as simply a function of cell-autonomous oncogenic mutations and the signaling events they perturb. Most of us have failed to fully appreciate that solid tumors are diseased tissues and that complex multicellular dynamics and interactions between malignant cells and the tumor microenvironment (TME) impact every aspect of disease etiology. To move the dial on cancer treatment, the TME must become a central consideration in the design of new cancer therapeutics.

How have we largely missed or undervalued this critical component for so long? Many of our experimental models were developed at a time when genetics dominated our thinking and were designed to test hypotheses about genetics rather than the TME. Most lack the substantial stromal infiltration and fibrosis that characterizes human disease. Consequently, new cancer drugs often undergo much of their early life being tested in models without a substantial TME, such as cell monocultures or rapidly growing clonal xenografts in immune-deficient mice. To genuinely incorporate the TME into drug development efforts will require a major shift in the way we think about tumors, from a focus on cell-autonomous molecular and biochemical pathways to an appreciation of multicellular systems. We must perform detailed comparative studies to determine whether, which and how our preclinical models recapitulate the TME of human disease.

**Fig 1:**
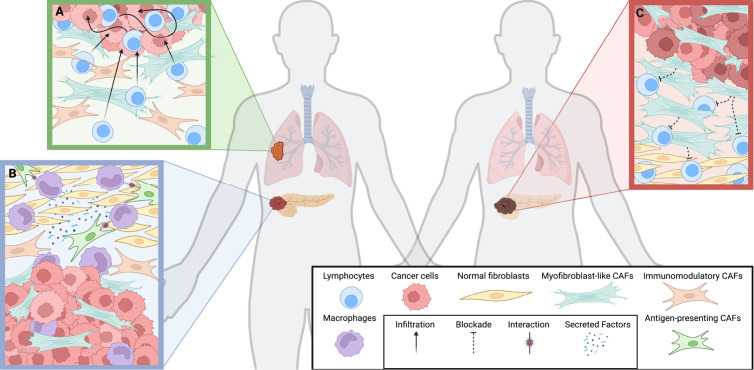
Context-dependent crosstalk between cancer associated fibroblasts and the tumor microenvironment. **A.** Lung tumor with tumor-infiltrating lymphocytes, with myofibroblast-like cancer-associated fibroblasts (myCAFs) adjacent to the tumor and inflammatory CAFs (iCAFs) at the tumor periphery. **B.** Pancreatic tumor with antigen-presenting CAFs (apCAFs) interacting with macrophages through direct cell–cell communication and secreted factors, promoting their polarization into a pro-inflammatory phenotype. **C.** Pancreatic tumor, dense with myCAFs adjacent to tumor cells, with a remodeled extracellular matrix that forms a physical barrier, impeding T cell infiltration and contributing to an immune-excluded phenotype. Created in BioRender.

The TME is a dynamic ‘ecosystem’ of cells in which neoplastic cells interact with mesenchymal and immune cells and extracellular molecules. Cancer-associated fibroblasts (CAFs) are central architects of this landscape. They deposit and remodel physical barriers through extracellular matrix (ECM) remodeling [1] that prevents entry of drugs and immune cells; secrete factors that nurture cancer cells; promote angiogenesis; and orchestrate immunosuppression [2,3]. However, the stroma, and the CAFs within it, are not invariant, passive entities. They exhibit remarkable heterogeneity, adopting diverse phenotypes that vary between organ sites (Fig 1) and treatment exposures; for example, chemotherapy can drive the emergence of a pro-fibrotic, CAF phenotype that promotes drug resistance and metastatic dissemination. This raises a crucial question: how do we develop a system to interpret stromal heterogeneity? Current efforts often focus on taxonomies based on surface markers, akin to immune cell classification. But given the profound plasticity of stromal populations in which cells transition dynamically between states, perhaps classification based on functions relevant to disease etiology and drug response—such as ECM deposition, immune modulation or antigen presentation—would be appropriate, much as we consider functional hallmarks of cancer cells.

Most stroma-targeting strategies in current use are based on a model of co-targeting, whereby stromal therapies are used to condition the tumor for greater impact by a conventional therapy, such as cytotoxic chemotherapy or immunotherapy. These strategies typically aim to target signaling from stromal cells through the use of antibodies or small molecule inhibitors (e.g., bevacizumab in the treatment of colorectal cancer or non-small cell lung cancer [4]), or to prevent or reverse the deposition and remodeling of dense ECM (e.g., pan-lysyl oxidase inhibitors as a novel therapy in pancreatic ductal adenocarcinoma (PDAC) [5]). In these co-targeting models, we must recognize the TME as a moving target, evolving through disease progression, metastasis and under therapeutic pressure. This dynamism demands careful consideration of the specific stromal state we aim to target. This is illustrated by the divergent impact of stromal Hedgehog pathway inhibition in breast cancers, where it sensitized mouse models and patients to cytotoxic chemotherapy and extended survival [6], versus pancreatic cancer, where it led to stromal depletion and worse outcomes [7]. Stromal therapies must therefore be carefully designed to achieve a net anti-tumor outcome.

Stromal reprogramming may have significant potential in enhancing the impact of immune therapies, particularly in so-called ‘immune cold’ tumors that are devoid of immune infiltrates. Emerging data suggests such tumors may have the potential to be immunogenic; however, nearby lymphocytes are rendered inactive by a hostile, exclusionary stroma. Evidence that tumor-killing T cells can be isolated even from immunotherapy-refractory diseases like PDAC [8] suggests that stromal intervention could unlock the efficacy of immunotherapies in these challenging contexts. Newer treatments like chimeric antigen receptor T (CAR-T) cells, which face significant hurdles in solid tumors due to stromal barriers and immunosuppressive signals, may also benefit from stromal co-targeting.

These challenges necessitate a radical rethinking of cancer drug discovery and development. Much earlier in drug development we must appreciate a drug’s impact on, or modulation by, the TME, such as inducing T-cell apoptosis or promoting fibrosis. Similarly, we should consider TME-derived signals that may bypass drug efficacy, like stromal-derived NRG1 conferring resistance to KRAS inhibitors in pancreatic cancer [9]. We need to develop new or modified models of disease that incorporate relevant components of the ECM and evaluate early drug candidates for their impact on the TME, much as we screen for toxicity against normal cells. This may not only avoid unintended consequences once agents enter trials but may identify dual-action drugs that can both directly target neoplastic or immune cells while also reprogramming the TME to promote their activity.

To capitalize on the opportunity that the TME affords for improvements in cancer treatment, several steps are necessary. We need to develop and validate more representative models, investing in preclinical models that faithfully recapitulate human TME complexity. Functional stromal classification needs to be embraced to enable us to move beyond static cell states and better understand dynamic stromal states, their functions and the molecular targets we can use to control them. We should aim to integrate TME assessment into drug development, enabling us to evaluate drug effects on and by the TME from the earliest stages. Similarly, we need to design smart, adaptive clinical trials that incorporate TME biomarkers and patient stratification for stromal therapies. To make all of this happen, we need to foster interdisciplinary collaboration and unite diverse expertise to tackle this multifaceted challenge.

We can draw parallels to immunotherapy’s early days, where broad, often poorly tolerated treatments gradually gave way to highly specific and transformative therapies through detailed cellular and functional classification and the maturation of the field of cancer immunology. A similar journey of careful analysis and functional understanding of the stroma, along with multidisciplinary science holds the potential to revolutionize cancer treatment.

The next generation of effective cancer therapies will be supported by our ability to understand and manipulate tumor ecosystems. By committing to this paradigm shift, we can cultivate a less hospitable ‘soil’, overcome resistance and unlock the full potential of our therapeutic repertoire.

## Abbreviations

CAFs: cancer-associated fibroblasts
CAR-T: chimeric antigen receptor T
ECM: extracellular matrix
PDAC: pancreatic ductal adenocarcinoma
TME: tumor microenvironment

## References

[1] Cox TR. The matrix in cancer. Nat Rev Cancer. 2021;21(4):217–38. doi: 10.1038/s41568-020-00329-7 . 33589810

[2] Linares J, Marín-Jiménez JA, Badia-Ramentol J, Calon A. Determinants and functions of CAFs secretome during cancer progression and therapy. Front Cell Dev Biol. 2021;8:621070. doi: 10.3389/fcell.2020.621070 . 33553157 PMC7862334

[3] Mao X, Xu J, Wang W, Liang C, Hua J, Liu J, et al. Crosstalk between cancer-associated fibroblasts and immune cells in the tumor microenvironment: new findings and future perspectives. Mol Cancer. 2021;20(1):131. doi: 10.1186/s12943-021-01428-1 . 34635121 PMC8504100

[4] Garcia J, Hurwitz HI, Sandler AB, Miles D, Coleman RL, Deurloo R, et al. Bevacizumab (Avastin^®^) in cancer treatment: a review of 15 years of clinical experience and future outlook. Cancer Treat Rev. 2020;86:102017. 32335505 10.1016/j.ctrv.2020.102017

[5] Chitty JL, Yam M, Perryman L, Parker AL, Skhinas JN, Setargew YFI, et al. A first-in-class pan-lysyl oxidase inhibitor impairs stromal remodeling and enhances gemcitabine response and survival in pancreatic cancer. Nat Cancer. 2023;4(9):1326–44. doi: 10.1038/s43018-023-00614-y . 37640930 PMC10518255

[6] Cazet AS, Hui MN, Elsworth BL, Wu SZ, Roden D, Chan C-L, et al. Targeting stromal remodeling and cancer stem cell plasticity overcomes chemoresistance in triple negative breast cancer. Nat Commun. 2018;9(1):2897. doi: 10.1038/s41467-018-05220-6 . 30042390 PMC6057940

[7] Lee JJ, Perera RM, Wang H, Wu D-C, Liu XS, Han S, et al. Stromal response to Hedgehog signaling restrains pancreatic cancer progression. Proc Natl Acad Sci U S A. 2014;111(30):E3091–100. doi: 10.1073/pnas.1411679111 . 25024225 PMC4121834

[8] Meng Q, Xie S, Gray GK, Dezfulian MH, Li W, Huang L, et al. Empirical identification and validation of tumor-targeting T cell receptors from circulation using autologous pancreatic tumor organoids. J Immunother Cancer. 2021;9(11):e003213. doi: 10.1136/jitc-2021-003213 . 34789550 PMC8601084

[9] Han J, Xu J, Liu Y, Liang S, LaBella KA, Chakravarti D, et al. Stromal-derived NRG1 enables oncogenic KRAS bypass in pancreas cancer. Genes Dev. 2023;37(17–18):818–28. doi: 10.1101/gad.351037.123 . 37775182 PMC10621596

